# X-linked Charcot Marie Tooth mutations alter CO_2_ sensitivity of connexin32 hemichannels

**DOI:** 10.3389/fncel.2023.1330983

**Published:** 2023-12-21

**Authors:** Jack Butler, Nicholas Dale

**Affiliations:** School of Life Sciences, University of Warwick, Coventry, United Kingdom

**Keywords:** connexin, Schwann cell, myelin, CMTX, neuropathy, hemichannel

## Abstract

Connexin32 (Cx32) is expressed in myelinating Schwann cells. It forms both reflexive gap junctions, to facilitate transfer of molecules from the outer to the inner myelin layers and hemichannels at the paranode to permit action potential-evoked release of ATP into the extracellular space. Loss of function mutations in Cx32 cause X-linked Charcot Marie Tooth disease (CMTX), a slowly developing peripheral neuropathy. The mechanistic links between Cx32 mutations and CMTX are not well understood. As Cx32 hemichannels can be opened by increases in PCO_2_, we have examined whether CMTX mutations alter this CO_2_ sensitivity. By using Ca^2+^ imaging, dye loading and genetically encoded ATP sensors to measure ATP release, we have found 5 CMTX mutations that abolish the CO_2_ sensitivity of Cx32 hemichannels (A88D, 111–116 Del, C179Y, E102G, V139M). Others cause a partial loss (L56F, R220Stop, and R15W). Some CMTX mutations have no apparent effect on CO_2_ sensitivity (R15Q, L9F, G12S, V13L, V84I, W133R). The mutation R15W alters multiple additional aspects of hemichannel function including Ca^2+^ and ATP permeability. The mutations that abolish CO_2_ sensitivity are transdominant and abolish CO_2_ sensitivity of co-expressed Cx32^WT^. We have shown that Schwannoma RT4 D6P2T cells can release ATP in response to elevated PCO_2_ via the opening of Cx32. This is consistent with the hypothesis that the CO_2_ sensitivity of Cx32 may be important for maintenance of healthy myelin. Our data, showing a transdominant effect of certain CMTX mutations on CO_2_ sensitivity, may need to be taken into account in any future gene therapies for this condition.

## 1 Introduction

Connexin32 (Cx32) is expressed in oligodendrocytes and Schwann cells of the central and peripheral nervous systems, respectively. These cells generate the myelin sheath around the central and peripheral axons that is required for high speed saltatory conduction. Charcot Marie Tooth disease is a slowly developing peripheral neuropathy that involves loss of the integrity of peripheral myelin (Murakami et al., 1996). Typical symptoms include slowed peripheral conduction, peripheral numbness and tingling, muscle wasting and excessive arching of feet. There is an X linked version of this neuropathy (CMTX) that is associated with mutations of Cx32 (Bergoffen et al., 1993). Strong evidence indicates that CMTX is caused by loss of function of Cx32 (Shy et al., 2007; Sargiannidou et al., 2009): a CMTX phenotype is present in Cx32-null mice (Scherer et al., 1998); and this can be rescued by re-expression of Cx32 targeted only to Schwann cells (Scherer et al., 2005; Sargiannidou et al., 2015).

Connexin32 is a beta connexin and is present in Schwann cells both as a hemichannel and a gap junction. In the paranode Cx32 acts as a plasma membrane channel opening from the paranode to the extracellular space. The opening of Cx32 during action potential propagation allows the release of ATP from the paranode into the extracellular space (Nualart-Marti et al., 2013). However, Cx32 gap junctions are also present at the Incisures of Lantermann. Here, they form “reflexive” gap junctions to permit a fast radial pathway of intracellular diffusion from the outermost to the inner most layer of myelin that is estimated to be 10^6^ times faster than the circumferential pathway via the spiral of the myelin sheath (Balice-Gordon et al., 1998). Strikingly, radial diffusion in myelin is not reduced in Cx32^–/–^ mice (Balice-Gordon et al., 1998), suggesting other connexins are able to play this role. The other major connexin expressed in Schwann cells is Cx29 (also known as Cx31.3).

The effect of CMTX mutations on the permeability and gating properties of Cx32 hemichannels and gap junctions have been studied (Omori et al., 1996; Oh et al., 1997; Ressot et al., 1998; Abrams et al., 2000, 2001; Wang et al., 2004; Sargiannidou et al., 2009). Various effects have been reported such as a deficiency in the Ca^2+^ triggered opening of Cx32 hemichannels in R220Stop (Carrer et al., 2018). For some mutations there is evidence of altered permeability of the gap junction to molecules (Oh et al., 1997; Bicego et al., 2006), which could reduce permeation of intracellular signaling molecules such as IP_3_ or cAMP through the gap junction. Nevertheless, the precise mechanistic reasons for why particular Cx32 mutations lead to CMTX are not well understood. In this study we have chosen 14 mutations that are associated with CMTX that occur in different regions of the molecule, including the N-terminus (important for gating), the transmembrane regions TM2 and TM3, the cytoplasmic loop and the C-terminus. Ten of these mutations are deemed by multiple sources to be pathogenic, while the remaining four are currently of uncertain status with regard to the pathology of CMTX (Table 1).

**TABLE 1:** CMTX-associated mutations analyzed in this study.

Region	Mutation	Pathological?	References
N-terminus	L9F	Uncertain	Brozková et al., 2010
	G12S	Pathogenic	Bergoffen et al., 1993; Abrams et al., 2001; Wang et al., 2004
	V13L	Pathogenic	Bone et al., 1997; Wang et al., 2004
	R15W	Pathogenic	Abrams et al., 2001; Wang et al., 2004; Record et al., 2023
	R15Q	Pathogenic	Fairweather et al., 1994; Abrams et al., 2001; Wang et al., 2004; Record et al., 2023
TM2	L56F	Uncertain	Latour et al., 1997; Ressot et al., 1998
	A88D	–	Lu et al., 2017
	V84I	Uncertain	Rouger et al., 1997
Cytoplasmic loop	E102G	Pathogenic	Ressot et al., 1998; Abrams et al., 2003; Record et al., 2023
	111–116 Del	Uncertain	Ionasescu et al., 1995; Ressot et al., 1998; Bicego et al., 2006
TM3	W133R	Pathogenic	Wang et al., 2004; Record et al., 2023
	V139M	Pathogenic	Bergoffen et al., 1993; Record et al., 2023
Extracellular loop	C179Y	Pathogenic	Casasnovas et al., 2006; Record et al., 2023
C-terminus	R220Stop	Pathogenic	Fairweather et al., 1994; Bicego et al., 2006; Carrer et al., 2018; Record et al., 2023

The classification of pathological status is based on the OMIM database (https://www.omim.org/entry/304040). A88D is not currently present within the OMIM database.

Connexin32 is a beta connexin, closely related to Cx26 and Cx30. Hemichannels of these three connexins can be opened by changes in PCO_2_ at constant extracellular pH and normal physiological concentrations of extracellular Ca^2+^ (Huckstepp et al., 2010a; Dospinescu et al., 2019). The CO_2_ sensitivity of the beta connexins is imparted by the presence of a “carbamylation motif” and involves carbamylation of a specific lysine residue within this motif, which then interacts with an Arg/Lys residue on the neighboring subunit in the hexamer (Meigh et al., 2013; Dospinescu et al., 2019; van de Wiel et al., 2020; Nijjar et al., 2021). In Cx32 the critical residues are K124, which we hypothesize to be carbamylated and K104 in the neighboring subunit which could act as the interacting partner (Dospinescu et al., 2019). Cx32 differs from Cx26, the connexin in which the mechanism of CO_2_ sensitivity has been best studied, in requiring markedly higher concentrations of CO_2_ to open (an increase of PCO_2_ to 55 mmHg or greater) (Huckstepp et al., 2010a; Dospinescu et al., 2019). In Cx26, several mutations that cause non-syndromic hearing loss or the keratitis ichthyosis deafness syndrome (KIDS) also abolish CO_2_ sensitivity (Meigh et al., 2014; de Wolf et al., 2016; Cook et al., 2019). In this paper, we address whether a range of CMTX mutations might affect the CO_2_ sensitivity of Cx32. By using a range of assays (Ca^2+^ imaging, dye loading and imaging of ATP release), we show that several CMTX mutations abolish the CO_2_ sensitivity of Cx32, but others do not affect it. Our data suggests that loss of CO_2_ sensitivity of Cx32 in certain CMTX mutations should be investigated further as a potential contributing mechanism to the development of the pathology.

## 2 Materials and methods

### 2.1 Cx32 mutations

The Cx32 gene sequences were synthesized by Genscript and subcloned into the pCAG-GS mCherry vector prior to mammalian cell transfection. Plasmids were generated using Gibson assembly. DNA fragments were generated using PCR amplification with primers (IDT). The presence of the correct mutation was confirmed by DNA sequencing (GATC Biotech). The dnCx32 plasmid was cloned using successive Gibson assemblies to incorporate both K104A and K124R mutations. All Cx32 constructs were inserted upstream of mCherry, with a short 12 amino acid linker (GVPRARDPPVAT).

### 2.2 Cell culture and transfection

Parental HeLa DH cells (ECACC Cat# 96112022, RRID:CVCL_2483) were grown in Low-glucose Dulbecco’s Modified Eagle Medium (DMEM), supplemented with 10% fetal bovine serum, 50 μg/mL penicillin/streptomycin. HeLa DH cells that stably expressed Cx32 (gift from Dr K. Willecke) were cultured in a similar manner, but with puromycin to select the expressing cells.

RT4-D6P2T cells were obtained from ECACC (ECACC Cat# 93011415, RRID:CVCL_4006). Cell culture was carried out in a modified medium; Dulbecco’s Modified Eagle’s Medium (DMEM) modified to contain 4 mM L-glutamine, 4500 mg/L glucose, 1 mM sodium pyruvate, and 1500 mg/L sodium bicarbonate and supplemented with 10% FBS, 5% Penicillin-Streptomycin.

Parental HeLa DH cells were plated onto coverslips at a density of 7.5 × 10^4^ cells per well of a 6 well plate, and transiently transfected with the Cx32 expression constructs following the PEI Transfection Reagent protocol. Cells were transfected using a mixture containing 1 μg DNA and 3 μg PEI for 24 h and imaged 48 h after transfection. For transfection of dnCx32, cells (either the RT4-D6P2T, or HeLa cells stably expressing Cx32) were seeded at a known density (10^4^ cells per well). These cells were then transfected with dnCx32. Recordings were obtained 5–6 days post-transfection to allow co-assembly with endogenous Cx32^WT^ to occur.

### 2.3 Solutions used

Control (35 mmHg PCO_2_) aCSF: 124 mM NaCl, 3 mM KCl, 2 mM CaCl_2_, 26 mM NaHCO_3_, 1.25 mM NaH_2_PO_4_, 1 mM MgSO_4_, 10 mM D-glucose saturated with 95% O_2_/5% CO_2_, pH 7.4, PCO_2_ 35 mmHg.

A total of 70 mmHg aCSF: 73 mM NaCl, 3 mM KCl, 2 mM CaCl_2_, 80 mM NaHCO_3_, 1.25 mM NaH_2_PO_4_, 1 mM MgSO_4_, 10 mM D-glucose, saturated with ∼12% CO_2_ (with the balance being O_2_) to give a pH of 7.4 and a PCO_2_ of 70 mmHg, respectively.

High K^+^ aCSF: 77 mM NaCl, 50 mM KCl, 2mM CaCl_2_, 26 mM NaHCO_3_, 1.25 mM NaH_2_PO_4_, 1 mM MgSO_4_, 10 mM D-glucose saturated with 95% O_2_/5% CO_2_, pH 7.4, PCO_2_ 35 mmHg.

### 2.4 Ca^2+^ imaging and analysis

HeLa-DH cells were transfected with the desired pCAG-Connexin-mCherry construct as detailed in methods of transfection. These cells were then incubated in 2 ml DMEM containing 5 mM Fluo-4 AM (Invitrogen) dissolved in 2.5 μL of Pluronic-127 (Invitrogen) for 20 min. Cells were then washed in 2 ml serum free DMEM for 20 min. Coverslips were then placed in a perfusion chamber. Cells were perfused with control 35 mmHg aCSF until a stable baseline is reached and maintained, at which point the cells were perfused with hypercapnic 70 mmHg aCSF. Once a stable baseline is reached the cells were then again perfused with control 35 mmHg aCSF. Following loading with Fluo-4 AM cells were imaged by epifluorescence (Scientifica Slice Scope, Cairn Research OptoLED illumination, 60x water Olympus immersion objective, NA 1.0, Hamamatsu ImagEM EM-SSC camera, Metafluor software). Fluo-4 was excited using 470 nm LED, with fluorescent emission being recorded between 507 and 543 nm. The Cx32 constructs utilized here yielded fusion proteins with a C-terminal mCherry tag. mCherry was excited with the 535 nm LED, with emission being recorded between 570 and 640 nm.

Analysis of Ca^2+^ signals was performed in ImageJ. For cells that had loaded with Fluo-4 and were positive for mCherry, an ROI was manually drawn round the cell body and the median pixel intensity within the ROI measured for each image. The fluorescence pixel intensities (F) were normalized to a baseline period (F_0_), and the difference in F/F_0_ evoked by the CO_2_ stimulus measured for each cell. This change in fluorescence was measured by taking the median of the F/F_0_ baseline from the 60–120 s immediately before the CO_2_ stimulus and subtracting this from the median F/F_0_ value measured over 60–120 s during the stimulus. Statistical comparisons were performed considering each cell as an independent measurement. Five transfections were performed for each variant of Cx32.

### 2.5 Dye loading assay and analysis

We used a dye loading protocol that has been developed and extensively described in our prior work (Dospinescu et al., 2019). HeLa cells expressing each Cx32 construct were initially washed with control solution. They were then exposed to either control or hypercapnic solution containing 200 μM 5(6)-carboxyfluorescein (CBF) for 10 min. Subsequently, cells were returned to control solution with 200 μM CBF for 5 min, before being washed in control solution without CBF for 30 min to remove excess extracellular dye. A replacement coverslip of HeLa cells was used for each condition. For each coverslip, mCherry fluorescence was imaged to verify Cx32 expression. The experiments were replicated independently (independent transfections) at least five times to give *n* = 5 for each species.

Following dye loading, HeLa cells were imaged by epifluorescence (Scientifica Slice Scope, Cairn Research OptoLED illumination, 60x water Olympus immersion objective, NA 1.0, Hamamatsu ImagEM EM-CCD camera, Metafluor software). Following acquisition of the images, subsequent analysis was performed blind to Cx32 variant and treatment. ImageJ was used to measure the extent of dye loading by drawing a region of interest (ROI) around each cell, and subsequently, the mean pixel intensity of the ROI was determined. The mean pixel intensity of a representative background ROI for each image was subtracted from each cell measurement from the same image. At least 40 cells were measured for each condition per experiment, and at least five repetitions of independently transfected HeLa cells were completed. Statistical comparisons were performed on the median values obtained from each transfection.

### 2.6 Measurement of ATP release and analysis

pDisplay-GRAB_ATP1.0-IRES-mCherry-CAAX was a gift from Yulong Li (Addgene plasmid #167582; RRID:Addgene_167582).^1^

pDisplay-GRAB_ATP1.0 mut-IRES-mCherry-CAAX was a gift from Yulong Li (Addgene plasmid #167583; RRID:Addgene_157583).^2^

Following transfection with GRAB_ATP_ cells were imaged by epifluorescence (Scientifica Slice Scope, Cairn Research OptoLED illumination, 60x water Olympus immersion objective, NA 1.0, Hamamatsu ImagEM EM-SSC camera, Metafluor software). cpGFP was excited using 470 nm LED, with fluorescent emission being recorded between 507 and 543 nm. The Cx32 constructs utilized here yielded fusion proteins with a C-terminal mCherry tag. mCherry was excited with the 535 nm LED, with emission being recorded between 570 and 640 nm.

Analysis of GRAB_ATP_ signals was performed in ImageJ. For cells that expressed both the Cx32 variant and GRAB_ATP_, one ROI was drawn around the region of GRAB_ATP_ expression per cell and the median pixel intensity within the ROIs measured for each image. The fluorescence pixel intensities (F) were normalized to a baseline period (F_0_), and the difference in F/F_0_ (ΔF/F_0_) evoked by the CO_2_ stimulus measured for each cell. Use of the GRAB_ATP_ biosensor to detect ATP release via Cx32 was validated by demonstrating that an increase in fluorescence to CO_2_ or 50 mM KCl was only seen in HeLa cells that expressed both Cx32 and GRAB_ATP_ (Supplementary Figure 1). No fluorescence changes to CO_2_ or 50 mM KCl were seen with either GRAB_ATP_ alone or Cx32 plus GRAB_mut_, an ATP insensitive mutated control (Supplementary Figure 1). As the dose response for GRAB_ATP_ was approximately linear over the range 0–3 μM (Supplementary Figure 1), and most of the recorded changes in ATP concentration fell into this range, we converted changes of fluorescence evoked by 70 mmHg PCO_2_ and 50 mM KCl into ATP concentration by normalizing them to the ΔF/F_0_ produced by a 3 μM calibration dose of ATP in each experiment. Statistical comparisons were performed considering each cell as an independent measurement. Five transfections were performed for each variant of Cx32.

### 2.7 Immunocytochemical staining and imaging

Coverslips were first washed with PBS three times, before being fixed in 4% PFA for 30 min. Coverslips were then washed in PBS three times and blocked using PBS containing 4% BSA and 0.1% Triton X-100 for 24 h. Cx32 primary antibody (1:250 dilution, Thermo Fisher Scientific Cat# 13-8200, RRID:AB_2533037) in PBS containing 4% BSA and 0.1% Triton X-100 was added to coverslips and left to incubate, constantly moving, for 3 h at room temperature. Coverslips were then washed using PBS containing 0.1% Triton X-100 six times at 10 min intervals. Anti-mouse secondary antibody (1:250, Thermo Fisher Scientific Cat# A-11032, RRID:AB_2534091) in PBS containing 4% BSA and 0.1% Triton X-100 and added to coverslips and left to incubate, constantly moving, for 2.5 h. The secondary antibody was washed using PBS containing 0.1% Triton X-100 six times at 10 min intervals. Coverslips where then mounted inverted on glass slides using Fluorshield™ with DAPI mounting medium (Sigma-Aldrich, Cat# F6057).

Cells were subsequently imaged using the Zeiss-880 confocal LSM, specifically using the 488 and 561 nm lasers. FIJI software was used for further analysis.

### 2.8 Statistical presentation and analysis

All quantitative data are presented as box and whisker plots where the box represents the interquartile range, the bar represents the median, and the whiskers represent 1.5 times the interquartile range. Individual data points are superimposed. Statistical analysis was via the Kruskal Wallis one-way ANOVA (KW test) followed by pairwise Mann Whitney *U*-tests with correction for multiple comparisons via the false discovery method (Curran-Everett, 2000) with the maximum rate of false discovery set at 0.05. For analysis of the GRAB_ATP_ recordings in which the CO_2_ and 50 mM KCl stimuli were applied to the same cell, these data were considered to be paired. Comparisons of the amount of ATP released by each stimulus was therefore performed with the Wilcoxon Matched Pairs Signed Rank test. All pairwise tests were two sided and all calculations performed with GraphPad PRISM.

## 3 Results

### 3.1 CMTX mutations alter CO_2_-dependent changes in Ca^2+^ influx via Cx32 hemichannels

**FIGURE 1:**
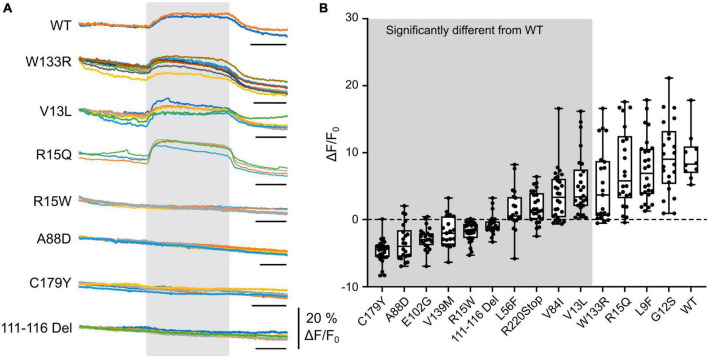
CO_2_-evoked increases in intracellular Ca^2+^ are mediated via Cx32 and sensitive to CMTX mutations. **(A)** Changes in Fluo4 fluorescence evoked by an increase of PCO_2_ from 35 to 70 mmHg (during gray rectangle) in HeLa cells expressing Cx32 WT and various CMTX mutations. Each colored line represents a recording from a different cell. Time scale bar 200 s. **(B)** Summary data for 14 CMTX mutations and WT Cx32, ordered from left to right by median change in fluorescence to CO_2_. A Kruskal Wallis ANOVA shows that these samples are not drawn from the same population (*p* < 0.0001). Pairwise testing relative to WT Cx32 (corrected for a maximum false discovery rate of 0.05) show that the mutations from the left up to and including V13L are significantly different from the WT (details in text).

To test whether CMTX mutations might alter the CO_2_ sensitivity of Cx32, we first used Fluo4 to measure intracellular Ca^2+^ in HeLa cells expressing WT Cx32. We found that a change in PCO_2_ from 35 to 70 mmHg reliably evoked a change in Fluo4 fluorescence (Figures 1A, B). This was not seen in parental HeLa cells that did not express Cx32 (Supplementary Figure 2). Thus, Cx32 hemichannels are permeable to Ca^2+^. A similar permeability to Ca^2+^ has previously been reported for Cx26 hemichannels (Fiori et al., 2012).

We next selected a panel of mutations that affected different regions of the Cx32 subunit, including the N-terminus (important in channel gating) and the cytoplasmic loop (the location of the carbamylation motif) and various transmembrane regions. Out of these 14 selected mutations, 10 affected the CO_2_-evoked Ca^2+^ signal via Cx32 (Figure 1). While 4 mutations (L56F, R220Stop, V84I (all *p* < 0.0001 compared to WT) and V13L, *p* = 0.0042 compared to WT) caused a partial reduction of the Ca^2+^ signal, the remainder (covering all portions of the molecule) caused an apparent complete loss of CO_2_ -dependent Ca^2+^ signal (Figure 1, *p* < 0.0001 compared to WT). Interestingly, the mutation R15Q had no effect on the CO_2_ mediated increase in intracellular Ca^2+^ whereas R15W caused its complete abolition. We have previously demonstrated in Cx26 that introduction of large residues at the N terminus (N14K and N14Y) abolished its CO_2_ sensitivity (de Wolf et al., 2016).

### 3.2 CMTX mutations alter CO_2_-dependent dye loading via Cx32

**FIGURE 2:**
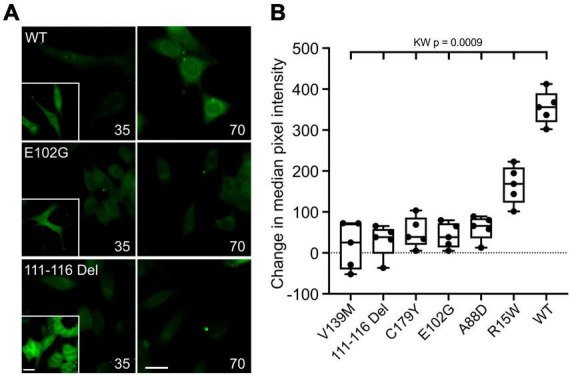
CMTX mutations in Cx32 can alter CO_2_-dependent dye loading. **(A)** Example images for HeLa cells expressing Cx32^WT^, Cx32^E102G^, and Cx32^111–116 Del^ of dye loading at 35 and 70 mmHg. The insets are a positive control showing dye loading in response to removal of extracellular Ca^2+^. Scale bars 20 μm. **(B)** Summary data for the 6 CMTX mutations that completely abolished CO_2_-evoked Ca^2+^ signals in HeLa cells, ordered by median change from left to right. A Kruskal Wallis ANOVA shows that these are not drawn from the same population. All the mutations are significantly different from the WT. While 5 of the mutations cause a complete loss of CO_2_-dependent dye loading, the effect of R15W is only partial.

An alternative interpretation of the above results is that some of these mutations might alter Ca^2+^ permeability of Cx32 rather than its sensitivity to CO_2_. We therefore further checked the effects of the six CMTX mutations that appeared to completely abolish CO_2_ sensitivity of Cx32, by using an established dye loading assay of hemichannel gating (Meigh et al., 2013; de Wolf et al., 2017; Dospinescu et al., 2019). We found that the mutations V139M, 111-116 Del, C179Y, E103G, and A88D completely abolished CO_2_-dependent dye loading (Figure 2) in agreement with the results from the Ca^2+^ measurements. This was not because there were no functional hemichannels, because the positive control of removing extracellular Ca^2+^ to unblock the hemichannels, gave robust dye loading for all 6 mutations (Figure 2 and Supplementary Figure 3). By contrast the mutation R15W only partially reduced the extent of CO_2_ dependent dye loading (Figure 2). Because the zero Ca^2+^ stimulus still gave robust dye loading (Supplementary Figure 3) the reduced dye loading in response to CO_2_ suggests a direct effect of R15W on CO_2_ sensitivity of the hemichannel. Nevertheless, the apparent complete abolition of a response to CO_2_ in the Ca^2+^ measurements indicates that this mutation may also greatly reduce the permeability of Cx32 hemichannels to Ca^2+^.

### 3.3 CMTX mutations alter CO_2_-dependent release of ATP via Cx32

**FIGURE 3:**
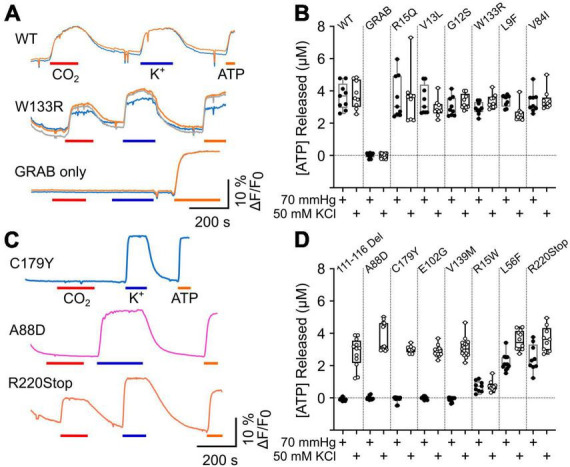
CMTX mutations in Cx32 can alter CO_2_ dependent ATP release. **(A)** Traces of changes in GRAB_ATP_ fluorescence to 70 mmHg PCO_2_ (red bar), 50 mM KCl (blue bar) and 3 μM ATP (calibration, orange bar) for HeLa cells expressing Cx32^WT^, Cx32^W133R^, and just GRAB_ATP_. **(B)** Summary data for CMTX mutations that do not alter CO_2_ sensitivity of Cx32 hemichannels. **(C)** Traces of changes in GRAB_ATP_ fluorescence to 70 mmHg PCO_2_ (red bar), 50 mM KCl (blue bar) and 3 μM ATP (calibration, orange bar) for HeLa cells expressing Cx32^C179Y^, Cx32^A88D^, and Cx32^R220Stop^. **(D)** Summary data for the CMTX mutations that alter CO_2_-dependent ATP release. Filled circles indicate ATP release to the CO_2_ stimulus, and open circles to the depolarizing stimulus in **(B,D)**.

The gating of connexin hemichannels has several developmental and physiological roles e.g., (Weissman et al., 2004; Pearson et al., 2005; Huckstepp et al., 2010b; Moore et al., 2014; van de Wiel et al., 2020). In many instances, hemichannel opening permits release of ATP which then mediates these physiological effects via P2 receptors. We therefore examined whether CMTX mutations altered CO_2_-dependent release of ATP via Cx32 hemichannels measured by co-expression of GRAB_ATP_. As the mutations might themselves alter the permeability to ATP, we used membrane depolarization (50 mM K^+^) as a positive control to trigger hemichannel opening independently of changes in PCO_2_. As might be expected Cx32^WT^ expressing HeLa cells released ATP in response to both CO_2_ and 50 mM K^+^ (Figures 3A, B). If HeLa cells were transfected only with GRAB_ATP_ no release of ATP was evoked by either stimulus (Figures 3A, B). Six CMTX mutations (R15Q, V13L, G12S, W133R, L9F, and V84I) gave ATP release to 70 mmHg PCO_2_ that was not significantly different from Cx32^WT^ (Figures 3A, B). However, the mutation L9F appeared to slightly reduce the voltage sensitivity of the hemichannel, as significantly less ATP was released by 50 mM KCl than Cx32^WT^ (*p* = 0.0028, Figures 3A, B). By contrast, the mutations 111–116 Del, A88D, C179Y, E102G, and V139M completely abolished CO_2_ dependent ATP release (all *p* = 0.0001 compared to Cx32^WT^) but did not affect the release of ATP evoked by 50 mM K^+^, suggesting a selective abolition of CO_2_ sensitivity in these mutants (Figures 3C, D). The mutations L56F and R220Stop had apparently normal depolarization evoked ATP release (compared to Cx32^WT^), but reduced CO_2_ dependent release suggesting a partial effect of these mutations on CO_2_ sensitivity (*p* = 0.0001 and *p* = 0.0078 compared to Cx32^WT^, respectively, Figures 3C, D). The mutation R15W very greatly reduced both CO_2_- and depolarization-evoked ATP release compared to Cx32^WT^ (*p* = 0.0001 and *p* < 0.0001, respectively, Figures 3C, D). The simplest interpretation is that permeability of the hemichannel to ATP release is greatly reduced but we cannot exclude additional effects of this mutation on voltage sensitivity or CO_2_ sensitivity, the latter being supported by the dye loading results.

We also compared the amount of ATP released from the CO_2_ stimulus and the depolarizing stimulus for each variant of Cx32. For the WT, V13L, W133R, V84I, R15Q, and R15W the amount of ATP released by the two stimuli was not significantly different. As might be expected from causal inspection of Figure 3, for the mutations 111–116 Del (*p* = 0.0005), A88D (*p* = 0.0001), C179Y (*p* = 0.002), E102G (*p* = 0.0005), V139M (*p* < 0.0001), L56F (*p* = 0.0005), and R220XStop (*p* = 0.0391) CO_2_ triggered significantly less ATP release than the depolarizing stimulus. For the mutation G12S, which was difficult to express, CO_2_ also caused slightly less ATP release compared to depolarization (*p* = 0.0117) whereas for L9F, CO_2_ caused slightly more ATP release compared to depolarization (*p* = 0.0273).

### 3.4 Characterization of a dominant negative Cx32 subunit (dnCx32)

Previously, we have generated a dominant negative subunit of Cx26 (dnCx26) by mutating the two residues involved in binding CO_2_ in that connexin (R104 and K125) (van de Wiel et al., 2020). As expected, dnCx26 is not sensitive to CO_2_. However, dnCx26 subunits can coassemble with those of WT Cx26 and remove CO_2_ sensitivity from the resulting heteromeric hexamer. We have shown that this is an effective tool *in vivo* to demonstrate the key role of Cx26 in respiratory chemosensing (van de Wiel et al., 2020). As the equivalent residues in Cx32 are K104 and K124, we, respectively, mutated them to Ala and Arg, to produce an equivalent dominant negative subunit for Cx32 (Cx32^K104A,K124R^, or dnCx32). Interestingly, individual mutations of K104 and K124 occur in patients with CMTX (Bone et al., 1997; Williams et al., 1999; Wang et al., 2015; Fattahi et al., 2017).

**FIGURE 4:**
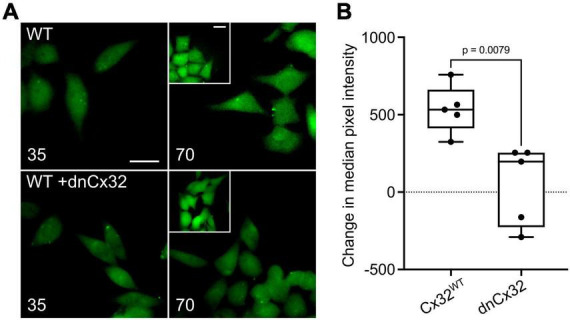
Characterization of a dominant negative Cx32 (dnCx32) that can remove CO_2_ sensitivity from Cx32^WT^. **(A)** Images of the HeLa cells stably expressing Cx32^WT^ (WT) with and without transient transfection of dnCx32, showing the loading of dye in response to increasing PCO_2_ from 35 to 70 mmHg. The inset shows the positive control of dye loading to a zero Ca^2+^ stimulus. Note that the transfection of the cells with dnCx32 abolishes CO_2_ dependent dye loading. Scale bars 20 μm. **(B)** Summary graph of the change in median pixel intensity in response to CO_2_. A total of 5 independent repeats for both the Cx32^WT^ and Cx32^WT^ + dnCx32 cells. Mann Whitney *U*-test, *p* = 0.0079 WT Cx32 vs. WT Cx32 + dnCx32.

We first used the dye loading assay to confirm that homomeric assemblies of dnCx32 are insensitive to CO_2_ (Supplementary Figure 4). We then found that when transfected into HeLa cells that stably expressed Cx32, the dnCx32 subunit was able to act in a dominant manner to abolish CO_2_-dependent dye loading (Figures 4A, B). As with dnCx26, it required 6 days of culture post-transfection for the dominant negative effect to become fully apparent.

### 3.5 dnCx32 blocks CO_2_-dependent dye loading and ATP release from Schwannoma cells

RT4 D6P2T Rat Schwannoma cells are a good model of Schwann cells and have previously been shown release ATP via the voltage-dependent opening of Cx32 hemichannels (Nualart-Marti et al., 2013). We therefore tested whether CO_2_ opened Cx32 hemichannels in this model system and whether this could be blocked by dnCx32 6 days after transfection.

**FIGURE 5:**
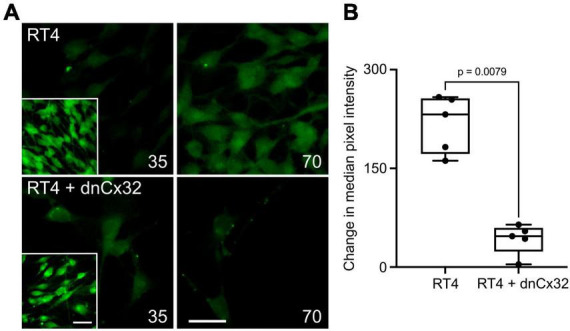
Cx32 mediates CO_2_-dependent dye loading in RT4 D6P2T Schwannoma cells. **(A)** Images of the Schwannoma cells loading with dye in response to increasing PCO_2_ from 35 to 70 mmHg. The inset shows the positive control of dye loading to a zero Ca^2+^ stimulus. Note that the transfection of the cells with dnCx32 abolishes CO_2_ dependent dye loading. Scale bars 20 μm. **(B)** Summary graph of the change in median pixel intensity in response to CO_2_. A total of 5 independent repeats for both the RT4 and RT4 + dnCx32 cells. Mann Whitney *U*-test, *p* = 0.0079 RT4 vs. RT4 + dnCx32.

Utilizing the dye-loading assay we demonstrated that the RT4 D6P2T cells robustly loaded with dye in response to both a zero Ca^2+^ challenge and an increase in PCO_2_ from 35 to 70 mmHg (Figure 5). However, transfection of the RT4 P6D2T cells with dnCx32 completely blocked their ability to load dyes in response to the CO_2_ challenge. Dye loading still occurred in response to the zero Ca^2+^ stimulus (Figure 5).

**FIGURE 6:**
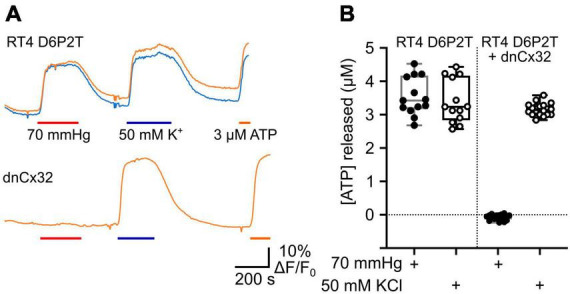
Cx32 mediates CO_2_-dependent ATP release from RT4 D6P2T Schwannoma cells. **(A)** Traces showing changes in GRAB_ATP_ fluorescence in response to a change in PCO_2_ (red bar), 50 mM KCl (blue bar) and 3 μM ATP (orange bar) for parental RT4 D6P2T cells and RT4 D6P2T cells transfected with dnCx32. Both sets of cells were tested after 7 days in culture. **(B)** Summary data showing that dnCx32 completely abolishes CO_2_-dependent ATP release (filled circles) from the RT4 D6P2T cells but does not affect their ability to release ATP to a depolarizing stimulus (50 mM KCl, open circles).

We also used co-expression of GRAB_ATP_ to measure ATP release from the RT4 D6P2T cells with and without expression of dnCx32 (Figure 6). In the parental RT4 D6P2T cells, 70 mmHg PCO_2_ and 50 mM KCl were equally effective at evoking ATP release (Figure 6). However, RT4 D6P2T cells that had been transfected with dnCx32 did not release ATP to the CO_2_ stimulus but displayed robust release of ATP to depolarization evoked by 50 mM KCl (Figure 6).

### 3.6 Transdominant effects of CMTX mutations on CO_2_ sensitivity of wild type Cx32

As Cx32 is expressed on the X chromosome, only one copy of the gene is ever expressed in a cell. For males this is obviously because they have only one X chromosome. However, in females one X chromosome is inactivated. In somatic tissues the selection of the X chromosome for inactivation occurs at random in the stem cell population and is conserved for all the subsequent progeny of the original parental cell (Riggs and Pfeifer, 1992). Thus, females will have stochastic and chimeric expression of their two X chromosomes. CMTX is generally less severe in females presumably because, if they are heterozygous for a CMTX mutation, sufficient Schwann cells will still express the wild type allele (Scherer et al., 1998). This means that potential transdominant effects of CMTX mutations have only been occasionally studied (Omori et al., 1996; Jeng et al., 2006). Nevertheless, in the context of genetic therapies, where expression of an additional wild type allele will be potentially used to cure the disease, transdominant effects may influence the outcome of such an intervention. Given that some syndromic mutations of Cx26 remove CO_2_ sensitivity and have a transdominant effect on the WT Cx26 allele (Meigh et al., 2014; de Wolf et al., 2016), at least some CMTX mutations could plausibly have a similar effect on the CO_2_ sensitivity of the WT Cx32 allele.

**FIGURE 7:**
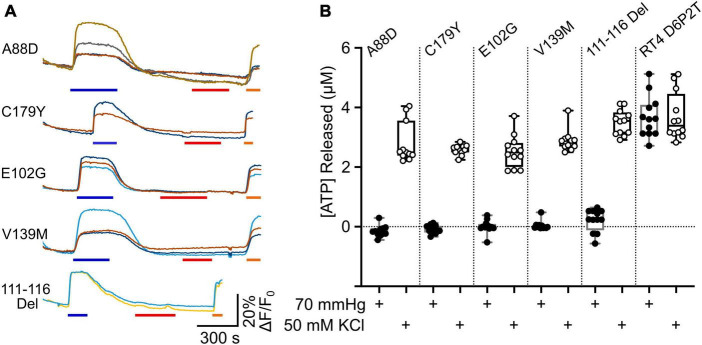
Transdominant effects of CMTX mutations on CO_2_ dependent ATP release from RT4 D6P2T cells. **(A)** Traces showing changes in GRAB_ATP_ fluorescence to 50 mM KCl (blue bar), 70 mmHg PCO_2_ (red bar) and 3 μM ATP (orange bar) for RT4 D6P2T cells expressing Cx32 carrying CMTX mutations that remove CO_2_ sensitivity. **(B)** Summary data showing the transdominant effect of these CMTX mutations on CO_2_ evoked ATP release (filled circles). The RT4 D6P2T cells express Cx32^WT^ which is able to release ATP both to depolarization (open circles) and an increase in PCO_2_ (see also Figure 6).

We expressed Cx32 with the mutations V139M, 111–116 Del, C179Y, E103G, and A88D in the RT4 D6P2T cells, to see whether these mutated subunits could remove CO_2_ sensitivity from the endogenously expressed Cx32^WT^. At the same time, we co-expressed GRAB_ATP_ to assay ATP release from these cells. We found that all 5 mutations completely prevented any CO_2_ dependent ATP release from the cells. However, in all 5 cases depolarization of the RT4 D6P2T cells with 50 mM K^+^ reliably evoked ATP release (Figure 7). This suggests that the mutant subunits co-assemble with Cx32^WT^ to make a heteromeric hexamer that is insensitive to CO_2_.

**FIGURE 8:**
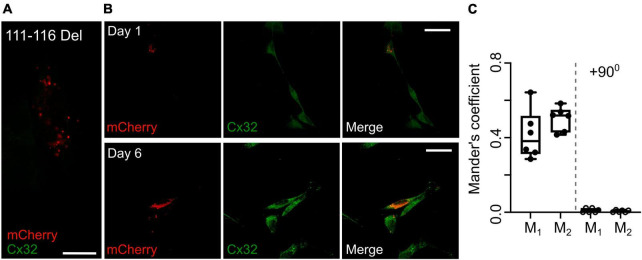
Expression of Cx32^111–116 Del^ does not alter expression of Cx32^WT^ in RT4 D6P2T cells. **(A)** Demonstration that the Cx32 antibody does not recognize Cx32^111–116 Del^. HeLa cells were transfected with this mutant tagged with mCherry and stained for Cx32: no staining is evident. Scale bar 15 μm. **(B)** Expression of Cx32^111–116 Del^ in RT4 D6P2T cells imaged 1 and 6 days after transfection. Note how the mCherry fluorescence colocalizes with the Cx32^WT^ expressed by these cells 6 days after transfection and visualized via the Cx32 immunostaining. **(C)** Analysis gives Mander’s coefficients that indicate substantial colocalization at day 6. The control was performed with one of the color channels rotated by 90^0^ with respect to the other. Each dot represents analysis performed on one RT4 D6P2T cell.

An alternative hypothesis is that expression of the mutant subunit suppresses expression of the endogenous wild type connexin. As our Cx32 antibody does not recognize the 111–116 Del mutant (Figure 8A), we stained RT4 D6P2T cells that coexpressed Cx32^111–116 Del^ and found that even after 6 days *in vitro* Cx32^WT^ was still expressed at levels that were indistinguishable the parental RT4 D6P2T cells and strongly colocalized with the mutant Cx32 as indicated by overlap of the immunofluorescence with that of the mCherry tag (Figures 8B, C). This supports our hypothesis that the transdominant action is exerted by coassembly of the mutant subunit into heteromeric hexamers.

## 4 Discussion

The key result we report is that a series of CMTX mutations affecting different regions of Cx32 abolish or greatly reduce CO_2_-dependent opening of the hemichannel. We used three different assays of hemichannel function: Ca^2+^ imaging to measure influx through Cx32; dye loading of a membrane impermeant fluorescent dye; and ATP release measured via GRAB_ATP_. All three assays provide results that indicate that the mutations 111–116 Del, A88D, C179Y, E102G, and V139M completely abolish CO_2_ sensitivity of the hemichannel. The Ca^2+^ imaging and ATP release assays both indicate that L56F and R220Stop have a partial effect on CO_2_ sensitivity. The Ca^2+^ imaging assays suggest that V84I and V13L have a small effect on the CO_2_-dependent Ca^2+^ influx. As these mutations have no effect on CO_2_-dependent ATP release, we suggest that this small effect does not arise from an alteration of the CO_2_ sensitivity of the hemichannel but may indicate a small alteration of Ca^2+^ permeability or a statistical fluke from multiple comparisons even although these are corrected to a maximum false discovery rate of 0.05. Overall, the consensus of our data is that 8/14 CMTX mutations tested (including R15W, discussed below) reduce or abolish the sensitivity of Cx32 hemichannels to CO_2_. All constructs in this study were tagged at the C-terminus with mCherry to allow visualization of expression. This C-terminal tag is unlikely to alter channel function [e.g., the CO_2_ sensitivity of tagged Cx32 described here seems identical to that of untagged Cx32 stably expressed in HeLa cells (Huckstepp et al., 2010a)], but we cannot exclude it might modify trafficking properties of some of the mutants.

CO_2_ dependent opening of Cx32 may have some physiological importance as we find that Cx32 acts as a CO_2_ sensitive conduit for ATP release from RT4 D6P2T cells, a rat Schwannoma cell line. We also found that the CMTX mutations that abolish CO_2_ sensitivity have a transdominant effect on Cx32^WT^ and abolish CO_2_ dependent ATP release from the Schwannoma cells.

The mutation R15W appears to have multiple effects on hemichannel properties: it alters Ca^2+^ permeability of the hemichannel (Figure 1) and most likely reduces ATP permeability (Figure 3). The dye loading data also suggest a substantial reduction in CO_2_ sensitivity as the fluorescent dye can still permeate the channel in the zero Ca^2+^ positive control for this assay (Figure 2 and Supplementary Figure 3). Our data cannot exclude that this mutation could also affect the voltage dependence of the hemichannel.

### 4.1 Comparison to mutations of Cx26

Mutations of Cx26 are the commonest cause of congenital non-syndromic sensorineural hearing loss. In addition, there are a small number of mutations that cause syndromic hearing loss. We have found that 4 of 9 mutations (A88V, N14K, N14Y, and A40V) that cause keratitis ichthyosis deafness syndrome abolish CO_2_ sensitivity of Cx26 gap junctions and hemichannels in a transdominant manner (Meigh et al., 2014; de Wolf et al., 2016; Cook et al., 2019; Nijjar et al., 2021). None of these mutations directly affect the CO_2_ sensing residues but are instead thought to decrease the flexibility of the molecule thereby preventing its reaction to changes in PCO_2_ (Brotherton et al., 2022). The effects on CO_2_ sensitivity for a range of CMTX mutations in Cx32 have striking similarities to the syndromic mutations we have studied in Cx26. Although the mutations are different, like Cx26 they do not affect the CO_2_ binding motif directly and they have transdominant effects on the CO_2_ sensitivity of Cx32^WT^. Given the similarity in sequence and structure of Cx32 to Cx26, it is plausible that the effects of the CMTX mutations on the CO_2_ sensitivity of Cx32 may also originate from the mutations restricting the flexibility of the molecule. It is important to note that the transdominant effects on CO_2_ sensitivity that we describe for some of the Cx32 mutations remain compatible with the strong evidence that shows CMTX being much less severe in females compared to males. As mentioned above, X chromosome inactivation ensures that in females each cell expresses genes from only one copy of the X chromosome (Gartler and Riggs, 1983; Lyon, 1988; Schwämmle and Schulz, 2023). For the same reason, as X chromosome inactivation occurs at random, in a nerve bundle of a heterozygous female (with one wild type and one mutant Cx32 allele) around half of the Schwann cells will express only the wild type gene and the remainder the mutant gene (Scherer et al., 1998). This presumably is enough to lessen the severity of CMTX in females compared to males carrying the same mutant (in whom, every Schwann cell will express the mutant gene).

### 4.2 Does loss of CO_2_ sensitivity of Cx32 contribute to CMTX?

As the symptoms of CMTX are recapitulated in Cx32-null mice and rescued by selective expression of Cx32 in Schwann cells, this progressive neuropathy is thought to arise from loss of Cx32 function. As 8/14 CMTX mutations tested cause partial or total loss of CO_2_ sensitivity, the question arises as to whether this could be a contributing mechanism to the etiology of CMTX. Furthermore, there are additional reports of CMTX-associated mutations affecting the CO_2_ sensing residues, K124 and K104 (Bone et al., 1997; Williams et al., 1999; Wang et al., 2015; Fattahi et al., 2017), which we predict (and indicated by dnCx32) would abolish CO_2_ sensitivity.

To examine whether there is any clinical evidence that supports loss of CO_2_ sensitivity as a contributor to CMTX, we have restricted our discussion to those mutations that are listed within the OMIM database^3^ as pathogenic and supported by evidence from multiple providers: R15Q, R15W, E102G, V139M, and R220Stop. Of these mutations R15W and V139M do not form gap junctions (Abrams et al., 2001). Our data show that R15W also lacks permeability to Ca^2+^ has greatly reduced permeability to ATP but may retain some reduced sensitivity to CO_2_. Given that these two mutations have multiple effects on the gap junction and hemichannel, they do not provide a test of whether loss of CO_2_ sensitivity is a sufficient contributor to CMTX. The mutations R15Q (Wang et al., 2004), E102G (Oh et al., 1997; Abrams et al., 2003) and R220Stop (Bicego et al., 2006) do not prevent gap junction formation. While R220Stop does cause a partial loss of CO_2_ sensitivity it also changes the sensitivity of Cx32 hemichannel-opening to intracellular Ca^2+^ (Carrer et al., 2018). Therefore, this mutation also cannot provide a test of our hypothesis. E102G stands out as a mutation that causes moderate severity CMTX while nevertheless forming gap junctions and, from the data reported here, having hemichannels with apparently normal voltage dependence and ATP permeability. Because E102G involves the loss of CO_2_ sensitivity of the hemichannel in the absence of other known functional effects on the hemichannel it lends some support to the hypothesis that the CO_2_ dependence of Cx32 may be important for the health of myelin. Set against this, is the fact that R15Q which has normal CO_2_ dependence, voltage dependence and ATP permeability and forms gap junctions, can cause CMTX. Almost certainly, there are likely to be several underlying mechanistic causes for CMTX, and loss of any one property of Cx32 may contribute to the origins of the neuropathy.

### 4.3 Potential therapeutic implications for CMTX

As CMTX is caused by lack of expression of functional Cx32 (e.g., defective trafficking mutants) or alterations of the properties of the mutant Cx32, gene therapy where a copy of the WT gene can be expressed to compensate for the missing function is a potential treatment under active development (Sargiannidou et al., 2015; Schiza et al., 2015; Kagiava et al., 2016, 2018, 2021a,2021b). In this context it is important to know whether the mutant copy of Cx32 could have transdominant effects as these will affect the success of the genetic therapy. Here we show that the CMTX mutations that abolish CO_2_ sensitivity also do this for RT4 D6P2T cells which express Cx32^WT^. If the CO_2_ sensitivity of Cx32 is a contributory factor in the development of CMTX, our data would suggest that expression of an additional copy of Cx32^WT^ may be an ineffective genetic treatment for these mutations.

## Data availability statement

The original contributions presented in the study are included in the article/Supplementary material, further inquiries can be directed to the corresponding author.

## Ethics statement

Ethical approval was not required for the studies on animals in accordance with the local legislation and institutional requirements because only commercially available established cell lines were used.

## Author contributions

JB: Conceptualization, Data curation, Investigation, Writing – review and editing. ND: Conceptualization, Supervision, Writing – original draft, Writing – review and editing.

## Footnotes

1
https://www.addgene.org/167582

2
https://www.addgene.org/167583

3
https://www.omim.org/entry/304040
